# Thromboelastometry-guided treatment algorithm in postpartum haemorrhage: a randomised, controlled pilot trial

**DOI:** 10.1016/j.bja.2022.10.031

**Published:** 2022-12-07

**Authors:** Samuli Jokinen, Anne Kuitunen, Jukka Uotila, Arvi Yli-Hankala

**Affiliations:** 1 Department of Emergency Medicine, Pain Medicine and Anaesthesiology, Tampere University Hospital, Tampere, Finland; 2 Department of Intensive Care, Tampere University Hospital, Tampere, Finland; 3 Department of Obstetrics and Gynaecology, Tampere University Hospital, Tampere, Finland; 4 Faculty of Medicine and Health Technology, Tampere University Hospital, Tampere, Finland

**Keywords:** coagulopathy, fibrinogen, massive haemorrhage protocol, obstetric anaesthesia, postpartum haemorrhage, thromboelastometry, transfusion

## Abstract

**Background:**

Postpartum haemorrhage causes significant mortality among parturients. Early transfusion of blood products based on clinical judgement and conventional coagulation testing has been adapted to the treatment of postpartum haemorrhage, but rotational thromboelastometry (ROTEM) may provide clinicians means for a goal-directed therapy to control coagulation. We conducted a parallel design, randomised, controlled trial comparing these two approaches. We hypothesised that a ROTEM-guided protocol would decrease the need for red blood cell transfusion.

**Methods:**

We randomised 60 parturients with postpartum haemorrhage of more than 1500 ml to receive either ROTEM-guided or conventional treatment, with 54 patients included in the final analysis. The primary outcome was consumption of blood products, and secondarily we assessed for possible side-effects of managing blood loss such as thromboembolic complications, infections, and transfusion reactions.

**Results:**

The median (25th–75th percentile) number of RBC units transfused was 2 (1–4) in the ROTEM group and 3 (2–4) in the control group (*P*=0.399). The median number of OctaplasLG® units given was 0 in both groups (0–0 and 0–2) (*P*=0.030). The median total estimated blood loss was 2500 ml (2100–3000) in the ROTEM group and 3000 ml (2200–3100) in the control group (*P*=0.033). No differences were observed in secondary outcomes.

**Conclusions:**

ROTEM-guided treatment of postpartum haemorrhage could have a plasma-sparing effect but possibly only a small reduction in total blood loss.

**Clinical trial registration:**

NCT02461251.

**Editor's key points**

• Postpartum haemorrhage remains a leading cause of maternal morbidity and mortality frequently requiring massive transfusion.
• This RCT compared goal-directed therapy with standard care in the treatment of postpartum haemorrhage.
• Thromboelastometry-guided transfusion therapy resulted in reduced plasma transfusion and estimated blood loss in this pilot study.
• A thromboelastometry-guided treatment protocol led to reduced use of fresh frozen plasma or OctaplasLG®, and possibly diminished bleeding amongst patients with severe postpartum haemorrhage compared with a conventional coagulation testing guided approach.
• Larger randomised and controlled studies are necessary to assess the effects on red cell administration, morbidity, and mortality.

Postpartum haemorrhage (PPH) remains among the leading causes of maternal mortality worldwide including in developed countries.^1^ Stepwise treatment protocols are warranted during this potentially catastrophic situation,^2^ and recent guidelines recommend monitoring of the coagulation status of the parturient and treating PPH in a goal-directed manner.^3^ As massive transfusion protocols (MTPs) consisting of early 1:1:1 transfusion of red blood cells (RBC), fresh frozen plasma (FFP), and platelets were considered advantageous for trauma patients,^4^ this treatment was adopted in the care of severe PPH,^5^ although scientific evidence was scarce.^6^ In addition, substantial variation exists between various society-derived guidelines,^7^ perhaps because administering large amounts of blood products is not without harm.^8^, ^9^, ^10^

Conventional coagulation tests, such as prothrombin time (PT), activated partial thromboplastin time (aPTT), and Clauss fibrinogen measurement, have limitations with regard to decision-making during active PPH: turnaround times are up to an hour and certain cut-off points for plasma transfusion based on PT and aPTT (usually 1.5 times the upper normal range) are derived from trauma settings.^11^ Fibrinogen is considered the first coagulation factor to decrease in PPH,^12^^,^^13^ and parturients with fibrinogen <2.0 g L^−1^ tend to progress to severe PPH, with a positive predictive value of 100%.^12^ With viscoelastic testing, such as rotational thromboelastometry (ROTEM®; Tem Innovations GmbH, Munich, Germany) the diagnosis of acquired hypocoagulation or hyperfibrinolysis is available earlier.^14^, ^15^, ^16^ It correlates with conventional coagulation tests during pregnancy.^17^ Patients having a ROTEM Fibtem A5 (the clot amplitude in mm at 5 min after the beginning of clot formation) equal to or under 12 mm at risk of being massively transfused, and with equal to or greater than 15 mm the progression of PPH is more unlikely.^18^ According to a recent Cochrane review, viscoelastic tests seem to be beneficial for patients with respect to mortality, morbidity, and diminished bleeding and blood product use, but most data consist of studies of cardiac surgery.^19^ Studies comparing these MTP-like or ‘shock pack’ strategies with ROTEM-guided protocols in PPH have been conducted recently,^20^^,^^21^ but these have been in a retrospective before–after setting. A prospective, randomised controlled study comparing standard and ROTEM-guided care was therefore warranted.

## Methods

This was a single-centre, single-blinded, randomised controlled trial. The protocol was approved by the ethics committee of Pirkanmaa Hospital District (PSHP) and conducted in its tertiary referral centre, Tampere University Hospital (Tampere, Finland; ethics committee reference number R15054). Registration of the protocol was made in May 2015 at Clinicaltrials.gov (NCT02461251). As postpartum blood loss is considered an emergency, written and informed consent was acquired by the study personnel as soon as possible after the operation to ensure the full legal capacity of the parturient and her next-of-kin following the principles of Word Medical Association's Declaration of Helsinki.

From January of 2016, women over age 18 yr with severe PPH (active bleeding of >1000 ml despite use of initial measures such as uterotonic agents and uterine massage after vaginal delivery thus requiring an intervention in the operating theatre or during/after Caesarean delivery) were included in the study.^22^ Bleeding disorders, such as known haemophilia or von Willebrand's disease, and refusal of allogeneic blood products were considered exclusion criteria. Patients were initially enrolled and randomised when they fulfilled these criteria to be treated according to a ROTEM-guided algorithm (ROTEM group) or standard care using conventional coagulation tests (control group). Randomisation (1:1 in blocks of 10) between groups was made beforehand by an independent statistician using a random number generator. The randomly assigned treatment protocol was put in a closed envelope, and after opening the topmost envelope on the pile located in the obstetrics operation department, the anaesthesiologist treating the patient followed the protocol assigned. The decision to recruit the patient depended not only on the eligibility criteria above but also on the anaesthesiologist's own judgement of their ability to follow the study protocol based on the urgency of the situation, resources available, and clinical experience.

**Fig 1:**
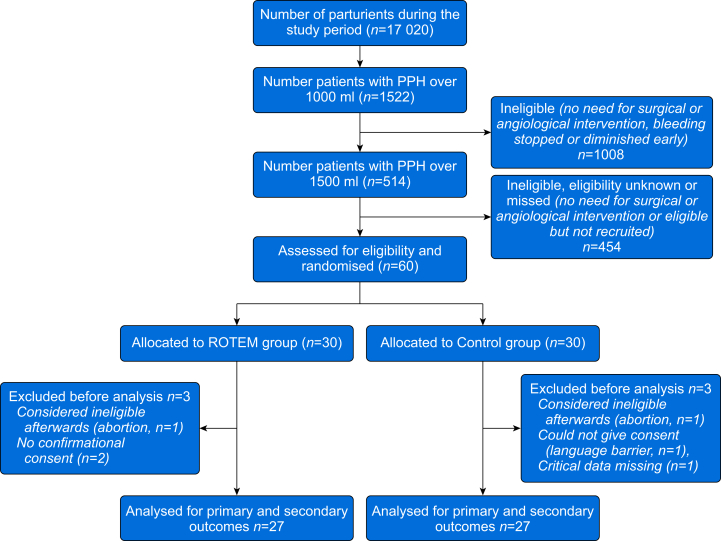
CONSORT diagram. CONSORT, Consolidated Standards of Reporting Trials; PPH, postpartum haemorrhage; ROTEM, rotational thromboelastometry.

The Consolidated Standards of Reporting Trials (CONSORT) diagram is presented in Fig. 1. Before starting to recruit patients, all anaesthesiologists treating obstetric patients received education on ROTEM in general and the study protocol. ROTEM measurements began at our institute in 2014 with ROTEM Delta® (Tem Innovations GmbH) being situated at the central laboratory with a real-time thromboelastogram monitor located in the obstetrics department, with the technique described elsewhere.^23^^,^^24^

After randomisation, initial blood samples were collected mainly through an arterial line including ROTEM Extem and Fibtem, blood count, aPTT, thromboplastin time as international normalised ratio (TT-INR), Clauss fibrinogen, and blood gas analysis with lactate measurement. During bleeding and postoperative surveillance, this testing was repeated if considered necessary by the anaesthesiologist, and 12–24 h after bleeding without arterial blood gas and lactate measurements. ROTEM measurements were also made in the control group but were not followed by the caregivers. The amount of blood loss was visually estimated before entering the operating theatre and after that, swabs and towels were weighed and the weight of dry items subtracted from the total to get blood weight; blood volume in suction canisters was directly measured and in Caesarean delivery, the estimated amount of amniotic fluid was subtracted from this figure.

**Fig 2:**
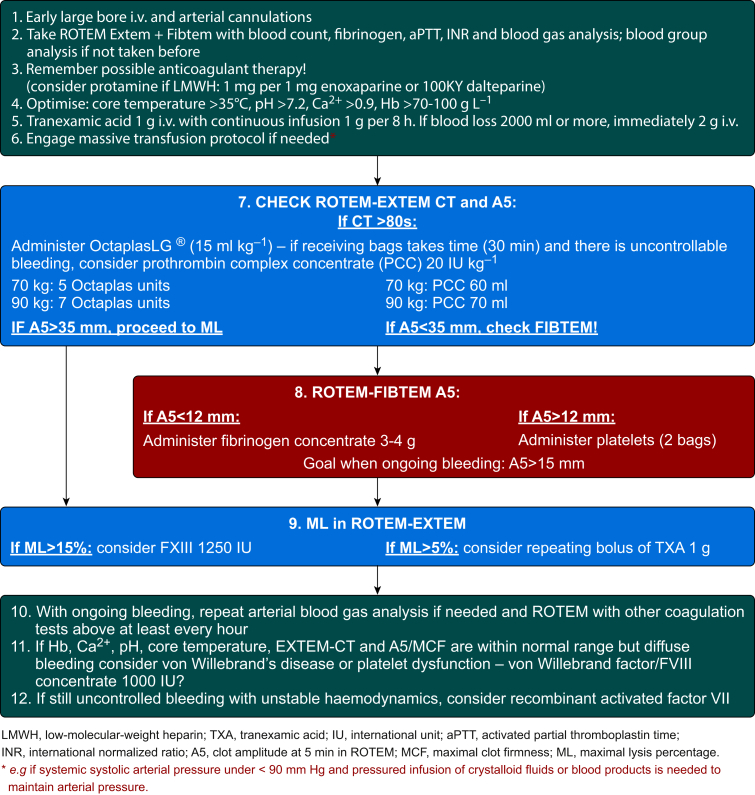
Intervention (ROTEM) group protocol. A5, clot amplitude at 5 min in ROTEM; aPTT, activated partial thromboplastin time; CT, clotting time; INR, international normalised ratio; IU, international unit; LMWH, low-molecular-weight heparin; MCF, maximal clot firmness; ML, maximal lysis percentage; ROTEM, rotational thromboelastometry; TXA, tranexamic acid. ∗For example, if systemic systolic arterial pressure under <90 mm Hg and pressured infusion of crystalloid fluids or blood products is needed to maintain arterial pressure.

**Fig 3:**
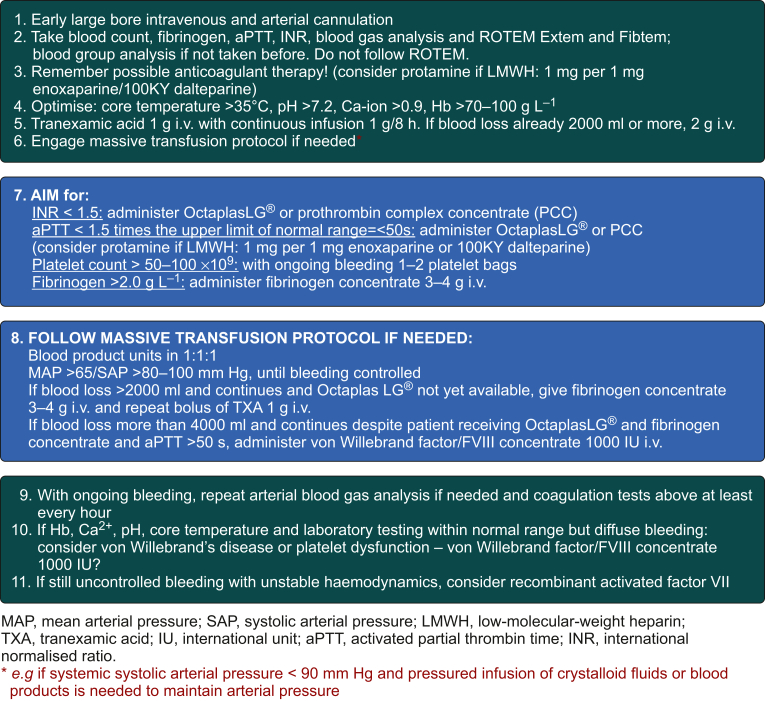
Control group protocol. aPTT, activated partial thrombin time; INR, international normalised ratio; IU, international unit; LMWH, low-molecular-weight heparin; SAP, systolic arterial pressure; TXA, tranexamic acid. ∗For example, if systemic systolic arterial pressure <90 mm Hg and pressured infusion of crystalloid fluids or blood products is needed to maintain arterial pressure.

The protocols for both groups are shown in Fig 2, Fig 3. Cut-off values were based on earlier data on normal peripartum reference ranges of ROTEM clotting time (CT),^25^ the usefulness of Fibtem A5 to predict MCF^16^^,^^18^^,^^26^ and possible worsening of PPH and contemporary European recommendations for treating perioperative haemorrhage adapted for our institute and for PPH.^27^ In case of increased TT or aPTT value or abnormal CT in ROTEM Extem, a pooled frozen plasma product, OctaplasLG® (Octapharma AB, Stockholm, Sweden), was the first-line therapy (rather than FFP). In severe cases with a delay in receiving plasma, a prothrombin complex concentrate (Cofact® [Sanquin Plasma Products B.V., Amsterdam, The Netherlands] or Octaplex® [Octapharma Pharmazeutika Produktionsgesellschaft m.b.H., Vienna, Austria]) could be used. In the ROTEM group, higher concentrations of fibrinogen were targeted during bleeding: a Fibtem A5 <12 mm was the trigger for administering fibrinogen concentrate (Riastap® [CSL Behring GmbH, Marburg, Germany] or Fibclot [LFB Biomedicaments, Lille, France]).^18^ In the control group, this was done on the basis of estimated ongoing blood loss >2000 ml and possibly later according to fibrinogen laboratory values, targeting >2.0 g L^−1^. A 1 g i.v. bolus of tranexamic acid (Caprilon®; Takeda Austria GmbH, Linz, Austria) with a sequential 1 g infusion in 8 h was recommended for all patients, but a bolus could be repeated if fibrinolysis was detected by ROTEM Extem; with more pronounced fibrinolysis (maximal lysis [ML], >15%), factor XIII concentrate (Cluvot®; CSL Behring GmbH) was to be added in ROTEM group.^28^^,^^29^ Infusions of von Willebrand factor/FVIII concentrate (Wilate®; Octapharma Pharmazeutika Produktionsgesellschaft m.b.H.) and activated recombinant factor VII (Novoseven®; Novo Nordisk A/S, Bagsværd, Denmark) concentrate were allowed in both groups under critical circumstances.

The anaesthesiologist was allowed to administer Ringer's acetate or albumin (Albuman®; Sanquin Plasma Products B.V.) to correct blood volume. Transfusion of blood products was not limited only according to haemoglobin or haemostatic measurements according to the protocols but also based on clinical judgement. The MTP (Fig. 3) could be initiated when suspected massive bleeding with unstable haemodynamics was present, for example SAP <90 mm Hg and pressurised infusion of crystalloid fluids or blood products needed to maintain blood pressure. Other treatment methods were at the discretion of the clinicians involved.

Participants were blinded. The attending anaesthesiologist and the anaesthetic nurse knew the assigned protocol, but the obstetric team assessing the amount of total blood loss was not informed. As for other outcome measurements, the assessor and the statistical analyst were blinded until the first draft of the manuscript. Data were collected with a case report form during and immediately after the bleeding but also afterwards through electronic patient records. Some information was tracked from manual patient files such as anaesthesia records. The number of transfused blood product units during the hospital stay was the primary endpoint; secondary endpoints were the number of transfusion reactions and thromboembolic complications within a 30-day period postpartum.

### Statistical analysis

For sample size calculation, the relevant information needed in this patient group was not available. In an earlier study, the median RBC given was 4 units.^20^ Assuming a mean of 3.0 transfused units and a standard deviation (sd) of 1.2 units, 24 patients per group would be needed to show a difference of 1 unit in the mean (alpha=0.05; beta=0.80). After adding a relevant missing ratio of 20%, we planned to enrol 30 patients in each group.

All analyses on outcomes were made with intention-to-treat and blinded. When not imminent, distributions of continuous variables were tested for normality using the Kolmogorov–Smirnov test for goodness-of-fit. If applicable, an independent samples *t*-test was used to compare the means of different variables between groups. The Mann–Whitney *U*-test was applied for information that did not fulfil criteria of parametric data. For binominal data, Pearson's χ^2^ test was applied, and if criteria for this were not fulfilled, Fisher's exact test was used and a two-tailed significance is reported. Analyses were made using IBM SPSS for Macintosh, version 25 (IBM, Armonk, NY, USA).

## Results

The first participant was recruited on February 8, 2016, and the study ended on September 2, 2019; a total of 60 patients were randomised and 54 subjects were included in the final analysis. Subjects' baseline characteristics are presented in Table 1 with no significant differences considering the possible risk factors for major PPH. The minimum total blood loss was 1500 ml.

**Table 1:** Baseline subject characteristics. For binominal variables, *P*-values are acquired using Pearson χ^2^ when applicable; otherwise, Fisher's exact test is used. One subject can have more than one (probable) cause of bleeding. Other trauma: vaginal rupture and ruptured sphincter. CD, Caesarean delivery; HELLP, haemolysis, elevated liver enzymes, and low platelets; IQR, inter-quartile range; LMWH, low-molecular-weight heparin; SAP, systolic arterial pressure; sd, standard deviation.

Variable	ROTEM (*n*=27)	Control (*n*=27)	*P*
Age at delivery, yr, mean (range)	32.8 (24–43)	31.5 (24–43)	0.341
BMI, kg m^−2^, mean (sd)	32.0 (6.42)	29.8 (6.52)	0.208
Weeks at delivery, median (range)	39.1 (25–41)	38.6 (30–42)	0.782
Vaginal births, median (range)	1 (0–3)	1 (0–8)	0.127
Caesarean deliveries, median (range)	1 (0–4)	0 (0–4)	0.373
Current delivery mode Vaginal, *n* (%) Caesarean delivery, *n* (%)	15 (55.6) 12 (44.4)	17 (63.0) 10 (37.0)	0.580
Duration of delivery (h), median (range)	7.15 (0.43–26.15)	7.75 (0.5–20.6)	0.883
Artificial fertilisation (%)	8 (29.6)	3 (11.1)	0.091
Multiple pregnancy, *n* (%)	1 (3.7)	4 (14.8)	0.351
Pre-eclampsia Mild, *n* (%) Serious, *n* (%) HELLP syndrome, *n* (%)	6 (22.2) 5 (18.5) 0 1 (3.7)	3 (11.1) 2 (7.5) 1 (3.7) 0	0.467
Abnormal placentation, *n* (%)	8 (29.6)	5 (18.5)	0.340
Chorioamnionitis	1 (3.7)	2 (7.4)	1
Induction of labour	8 (29.6)	11 (40.7)	0.393
Instrumented vaginal delivery	4 (14.8)	2 (7.4)	0.669
Oxytocin infusion antepartum	14 (51.9)	14 (51.9)	1
Platelet count (×10^9^), mean (sd)	206 (65)	187 (67)	0.295
Haemoglobin at admission to labour ward (g L^−1^), mean (sd)	121 (10.2)	120 (9.7)	0.560
Blood loss before entering theatre (ml), median (IQR)	600 (2000)	1500 (1500)	0.118
SAP <80 mm Hg (at any study point), *n* (%)	13 (48%)	16 (59%)	0.586
LMWH during pregnancy	2 (7.4)	2 (7.4%)	1
Cervical laceration	2 (7.4)	2 (7.4)	1
Uterine rupture	1 (3.7)	0	1
Other trauma of birth canal	3 (11.1)	3 (11.1)	1
Placental retention (total or partial)	12 (44.4)	10 (37.0)	0.580
Atony	8 (29.6)	12 (44.4)	0.260
Surgical bleeding only (in CD)	4 (14.8)	2 (7.4)	0.699

The primary endpoint data were available for 54 subjects. The median (25th–75th percentile) amount of transfused RBC units was 2 (1–4) in the ROTEM group and 3 (2–4) in control group (*P*=0.399). The number (%) of subjects transfused were 21 (77.8%) and 23 (85.2%), respectively (*P*=0.484). As for OctaplasLG® units only, the numbers of subjects transfused were 5 (18.5%) and 12 (44.4%) (*P*=0.040), respectively; the median amount was 0 units in both groups (0–0 in the ROTEM group, 0–2 in the control group; *P*=0.030). All OctaplasLG® transfusions were based on clinical judgement: it was part of a complete 1:1:1 blood product protocol with one subject in each group. In both groups, 3 subjects (11.1%) had a platelet transfusion but only 1 (in ROTEM group) had platelet count <50×10^9^ and abnormal ROTEM Extem A5. The total median blood loss was 2500 (2100–3000) ml in the ROTEM group and 3000 (2200–3100) ml in the control group (*P*=0.033).

Administered OctaplasLG® units, medications, and surgical treatment modalities of PPH are listed in Table 2. A significant difference was seen in sulprostone infusion use, which happened more often in the control group (2 *vs* 8 patients, *P*=0.036). No subject received prothrombin complex concentrate (PCC), factor XIII, or activated recombinant factor VII concentrate. Albumin was not used. An MTP was launched in one subject in each group with blood products given at a 1:1:1 ratio. One subject in the ROTEM group received blood products including platelets and fibrinogen concentrate despite normal Extem A5 and Fibtem A5. This is considered protocol violation, but the data on this subject were still included in the ROTEM group.

**Table 2:** Use of procoagulant and uterotonic medications and other methods in treatment of postpartum haemorrhage. sd, standard deviation. ∗ P-value < 0.05.

Treatment	ROTEM	Control	*P*-value
OctaplasLG®, *n* (%)	5 (18.5)	12 (44.4)	0.040∗
Fibrinogen concentrate, *n* (%) In g, median	4 (14.8) 0 (0–0)	7 (25.9) 0 (0–2)	0.311 0.343
Fibrinogen with OctaplasLG®, *n* (%)	3 (11.1)	5 (18.5)	0.444
Tranexamic acid, *n* (%) In mg, median (25–75th percentile)	27 (100) 2000 (1000–2000)	26 (96.3) 2000 (1150–2000)	1 0.200
Crystalloid fluid (ml), mean (sd)	4257 (1677)	3743 (1141)	0.194
Oxytocin infusion, *n* (%)	23 (85.2)	26 (96.3)	0.351
Misoprostole p.r., *n* (%)	13 (48.1)	16 (59.3)	0.413
Methylergometrin, *n* (%)	13 (48.1)	15 (55.6)	0.586
Sulprostone infusion, *n* (%)	2 (7.4)	8 (29.6)	0.036∗
Emergency hysterectomy, *n* (%)	2 (7.4)	0	0.491
Angiological procedure, *n* (%)	4 (14.8)	0	0.111
Bakri balloon, *n* (%)	6 (22.2)	7 (25.9)	1

Laboratory values measuring the quality of haemostatic and haemodynamic management, such as haemoglobin, platelet count, TT-INR, aPTT, lactate, base excess, and ROTEM Extem and Fibtem values were determined at the beginning of treatment and 12–24 h afterwards. These parameters were also measured during treatment if considered necessary by the anaesthesiologist. Only Extem Maximal Lysis measured 12–24 h after treatment showed a significant difference (Table 3).

**Table 3:** Key laboratory values at the beginning and 12–24 h after treatment. Clinically worse (lowest or highest) values during the study period and number of subjects exceeding protocol limits are also presented. Data expressed as mean (sd) for normally distributed variables or median with 25–75% IQR. For lowest or highest values, mean or median and range are presented. A5, clot amplitude at 5 min; aPTT, activated partial thromboplastin time (reference range, 23–35 s); CT, clotting time; IQR, inter-quartile range; MCF, maximal clot firmness; ML, maximal lysis; sd, standard deviation; TT-INR, thromboplastin time – international normalised ratio (reference range, 0.9–1.2).

Measurement	ROTEM	Control	*P*-value
Haemoglobin, (g L^−1^) Beginning After Lowest, mean (range) <70 g L^−1^, *n* (%)	94 (13.2) 91 (8.6) 86 (68–106) 1 (3.7)	96 (16.6) 92 (13.1) 88 (63–121) 3 (11.1)	0.633 0.650 0.464 0.610
TT-INR Beginning After Highest, median (range) >1.5, *n* (%)	1 (1–1.1) 1 (1–1.1) 1.0 (0.9–1.2) 0	1 (1–1.1) 1 (1–1.1) 1.1 (0.9–1.3) 0	0.448 0.300 0.434
Platelet count (×10^9^) Beginning After Lowest, mean (range) <50×10^9^, *n* (%)	179 (50) 181 (60) 163 (36–239) 1 (3.7)	168 (63) 147 (64) 141 (76–319) 0	0.484 0.052 0.116 1
aPTT (s) Beginning After Highest, median (range) >50 s, *n* (%)	30 (29–32) 32.5 (31–35) 32 (29–40) 0	30 (29–32) 34 (31–35) 32 (28–39) 0	0.380 0.300 0.837
Fibrinogen (g L^−1^) Beginning After Lowest (mean, range) <2.0 g L^−1^, *n* (%)	3,5 (0.8) 4.1 (0.8) 3.4 (2.0–4.8) 0	3.4 (0.8) 4.2 (0.6) 3.4 (1.8–5.3) 2 (7.4)	0.703 0.653 0.876 0.491
Extem CT (s) Beginning After Longest, mean (range) >80 s, *n* (%)	49 (6) 50 (6.6) 52 (42–65) 0	50 (6.2) 47 (6.5) 51 (40–62) 0	0.723 0.234 0.489
Extem A5 (mm) Beginning After Lowest, mean (range) <35 mm, *n* (%)	45 (7.8) 46 (7) 43 (31–55) 4 (14.8)	43 (8.8) 46 (6.6) 44 (30–56) 3 (11.1)	0.398 0.842 0.830 0.685
Extem MCF (mm) Beginning After Lowest, mean (range)	67 (6.3) 67 (5.3) 64 (52–78)	64 (10) 66 (4.4) 65 (56–72)	0.272 0.313 0.878
Extem ML (%) Beginning After Highest, mean (range) >15%, *n* (%)	5 (3) 6 (3.1) 6 (1–12) 0	4,6 (2.7) 8 (2.7) 7 (1–14) 0	0.604 0.011∗ 0.270
Fibtem A5 (mm) Beginning After Lowest, mean (range) <12 mm, *n* (%)	14 (5.2) 15 (4.4) 13 (6–23) 10 (37)	13 (3.6) 15 (3.7) 13 (6–20) 11 (40.7)	0.429 0.741 0.888 0.780
Fibtem MCF (mm) Beginning After Lowest, mean (range)	18 (6.2) 20 (5.9) 17 (9–29)	17 (4.3) 20 (4.7) 17 (9–25)	0.353 0.735 0.837
Ca^2+^ (mM) Lowest, mean (range) <0.90 mM, *n* (%)	1.16 (0.05) 1.13 (0.96–1.28) 0	1.18 (0.04) 1.12 (0.98–1.21) 0	0.076 0.866
Lactate (mM) Highest, median (range)	1.6 (1.0–2.1) 1.95 (0.3–6.0)	1.5 (1.1–1.9) 1.65 (0.7–5.9)	0.741 0.614
Base excess (mEq) Lowest, mean (range)	–5.5 (3.9) –6.0 (–12.8 to –0.2)	–4.8 (2.1) –5.6 (–11.5 to –1.9)	0.425 0.622

As a secondary endpoint, we assessed the number of complications possibly linked to treatment of haemorrhage. One transfusion-associated circulatory overload was noted in the ROTEM group. Fever was reported after transfusion in 4 (14.8%) subjects in each group. There were no reported thromboembolic complications. Two subjects (7.4%; endometritis, rupture of the incision) in the ROTEM group and 4 subjects (14.8%; pyelonephritis, bleeding, allergic reaction caused by antibiotics, mastitis) in the control group were admitted to hospital during the 30-day postpartum period (*P*=0.669). No statistically significant differences between groups were observed in the number of postpartum infections, ICU admissions, time spent in the PACU, or length of hospital stay. No maternal deaths occurred in the study population.

As a quality control, we monitored the blood product transfusion threshold during the study. This remained unchanged: among the first 15 consecutive patients recruited the median of total blood loss divided by number of RBC units administered was 1000 (762–1400), and the last 15 patients had a ratio of 825 (550–1050) (*P*=0.228). For OctaplasLG®, the respective figures were 2500 (1625–3000) and 1500 (1337–1969) (*P*=0.200).

## Discussion

Use of ROTEM® thromboelastometry might decrease administration of plasma by avoiding unnecessary OctaplasLG® transfusions. This finding agrees with similar studies conducted in other patient groups, such as those who underwent aortic^30^ and cardiac surgery,^31^ liver transplantation,^32^ and burn surgery.^33^ A recent observational study on parturients showed that by using ROTEM-derived parameters, FFP administration is often avoided.^34^ As for PPH, other non-randomised studies have given the same signal.^20^^,^^21^ Although in both groups blood component transfusion based on the clinical situation (e.g. suspected or measured haemodynamic instability or severe and rapid blood loss) was allowed, the number of subjects receiving OctaplasLG® was lower in the ROTEM group.

As experience from trauma patients suggested the benefit of a greater FFP/RBC ratio, there has been a tendency among clinicians to administer FFP at a lower threshold, even against current guidelines.^13^ Use of ROTEM might provide confidence to refrain from early plasma transfusions, as the test results are rapidly available, or might terminate MTP earlier after observing normal ROTEM measurements. The control protocol might be perceived as more liberal with regard to early plasma transfusion as it reflected the general guidelines for the treatment of major blood loss at our institute at the time. The clinical situation and the background for the decision to start transfusing OctaplasLG® with RBC varied in our study. Only two subjects received a complete ‘shock-pack’ of blood products, and 11 subjects had a recorded SAP under 80 mm Hg with continuous bleeding but did not receive a complete MTP ‘shock-pack’, whether because the bleeding had stopped or diminished or a normal Extem CT was noted. Two subjects in the control group had blood loss >2 L before entering the theatre, and therefore plasma transfusions were commenced empirically. One subject in the control group had a severe and rapid blood loss of 3 L in a 30-min procedure but no record of SAP <80 mm Hg. One subject in the ROTEM group received both plasma and platelets despite normal ROTEM measurements (owing to a power failure, the ROTEM values were not available).

Fibrinogen concentrate was also used more often (25.9% *vs* 14.8% of cases, *P*=0.311) in the control group. Although not statistically significant, this difference might result from not knowing the fibrinogen concentration or Fibtem A5 value (which has been shown to correlate with plasma fibrinogen concentration^16^^,^^20^) and administering fibrinogen concentrate only intuitively. In the control group, blood loss of >2 L was the trigger for 6 (22.2%) subjects, and 1 (3.7%) subject received fibrinogen because of low fibrinogen measurement. ROTEM Fibtem A5 values or Clauss fibrinogen concentrations did not differ significantly between groups. Pre-emptive use of fibrinogen has been shown to be futile in parturients,^35^ but at the time of our study design some guidelines still recommended its early use^22^^,^^27^^,^^36^ in major haemorrhage, especially if fibrinogen concentrations were not known. Therefore, it was included in the control group protocol. Fibrinogen concentration before parturition is perhaps not as good a prognostic marker for severe PPH as the concentration measured during bleeding.^12^^,^^37^^,^^38^ Our study also found no correlation between plasma fibrinogen concentration measured during PPH and total estimated blood loss either (Pearson's correlation coefficient=–0.156, *P*=0.260), but the concentration was <2.0 g L^−1^ only in two patients in the control group (*P*=0.491).

We did not find a significant difference in RBC transfusions as presumed, although the median number of RBC units administered was lower in the ROTEM group. When moving from the ‘shock-pack’ era towards more individualised care of bleeding, this might be expected but is seen also in randomised and prospective studies.^30^, ^31^, ^32^, ^33^ On the contrary, a recent observational study reported no benefit of ROTEM with regard to RBC transfusion in PPH.^39^ However, in our study a small but statistically relevant difference in median total blood loss in favour of the ROTEM group was seen. Whether this is a result of more efficient haemostatic resuscitation is uncertain. Similar results have been presented in other patient groups as stated in a recent Cochrane review.^19^

Other laboratory parameters measuring blood coagulation and adequacy of fluid therapy were comparable and within accepted limits. The difference in post-treatment Extem ML measurement (6% in the ROTEM group, 8% in the control group; *P*=0.011) might not be of clinical significance, as both values are within the reference range of <15%. This might represent the good quality of care in both groups, or the quality of subjects recruited being ‘too good’. As for the latter, 1:1:1 blood product transfusion was considered necessary only in one case in each group; thus massively bleeding patients were perhaps too few. Only one subject (in the ROTEM group) had a blood loss of >5 L.

Of interventions to control haemorrhage, only sulprostone infusion was more often frequent in the control group. Sulprostone is a synthetic prostaglandin E2 derivative and a prostaglandin E3 agonist, a second-line therapy used to control uterine atony,^3^ which was diagnosed in 29.6% in the ROTEM group and 44.4% in the control group (*P*=0.260). *In vitro*, sulprostone enhances aggregation of activated platelets but this effect might not be of clinical importance.^40^ Although the ROTEM group could have had better haemostatic resuscitation so not needing these secondary interventions, a more reasonable explanation might be a greater incidence of atony in the control group. Emergency hysterectomies and vascular interventions occurred more often in the ROTEM group but the differences were not significant.

To our knowledge, this is the only randomised controlled study among parturients comparing a ROTEM-guided treatment protocol with a conventional coagulation testing-guided approach. In this pragmatic study, the protocols were made simple enough to ensure implementation, patients were recruited no matter the day or time and by 22 different anaesthesiologists, and not only those involved in the study group. As this might have led to some heterogeneity in how subjects were treated and protocols followed, this is often the case even when using standard operating procedures.

Our study had several weaknesses. As the median blood loss was 2.5–3.0 L and 1:1:1 transfusion was seldom needed, we might have missed parturients with lack of fibrinogen or other situations where early diagnosis of hypocoagulation could change the course of haemorrhage. Also, in a true emergency with patients on the verge of cardiovascular collapse, perhaps combined with shortage of personnel, suitable patients might not have been recruited. The attending anaesthesiologist was primarily taking care of the patient, not the study and we had no study personnel to help during on-call hours. Some patients were also missed because of unawareness of the ongoing study. This might explain why the study period was longer than planned. However, the treatment protocols remained unchanged during the study period.

Subjects had a mean RBC transfusion amount of 2.6 units (sd, 1.67), which is less than our assumed amount; thus the sample size was insufficient. Despite this, some signals of diminished need for transfusions were seen in the ROTEM group. The difference in total blood loss between groups was small, and might not be as clinically as relevant as it seems because laboratory parameters and RBC needs did not differ significantly. Mean blood loss (2716 ml; 95% confidence interval [CI], 2366–3066 in the ROTEM group; and 2946 ml; 95% CI, 2712–3181 in the control group) was not significantly different (*P*=0.266). However, the data were not parametric and as this outcome was assessed and analysed blinded, it could still be interpreted as a true difference. However, blood loss before entering theatre was greater in the control group. Although this difference was not significant (*P*=0.118), it might affect the total blood loss. As the amount of haemorrhage on the labour ward was estimated visually in part, these figures must be interpreted with caution.

In conclusion, we provide evidence that viscoelastic testing leads to more reasonable use of FFP or OctaplasLG®, and possibly diminishes bleeding, amongst patients with severe PPH. Larger randomised and controlled studies that apply a greater bleeding threshold for inclusion are warranted to assess the effects on red cell administration, morbidity, and mortality.

## Authors' contributions

Recruitment of subjects: SJ, AY-H

Statistical analyses: SJ

Preparation of the first draft of the manuscript: SJ

Manuscript revision: AY-H, AK, JU

All authors participated in the study design and analysis of results, and read and approved the final version of the manuscript.
